# Classification in Board Schools

**Published:** 1898-07-30

**Authors:** 


### The Institutional Workshop.

### CLASSIFICATION IN BOARD SCHOOLS.

In an address delivered at the opening of a new board school at Bothwell, Mr. James Smith, one of H.M. Inspectors of Schools, made some interesting remarks on the different character of the education which is now required under the Education Acts compared with what used to be given in times gone by. The old Scottish schools were, he said, distinctly in advance of those found elsewhere, yet even in them, partly owing to deficiency in staff, and partly to the predilections of the teachers, the greater portion of their time was spent on the instruction of the most apt pupils, to the comparative neglect of the rest of the school. This system was completely revolutionised by the introduction of the much maligned "Revised Code," which enforced the teaching of every pupil, insisted on his progressive instruction, and caused the test of progress to be applied by the inspector at his annual visit. The result of the code was that the dull pupils received the greatest attention, while the brighter ones were now in comparison neglected. This revised code, was, no doubt, too inflexible, too cast-iron in its structure, and it has had to be modified repeatedly, but at least it must be allowed the credit of having indented into the mind of the country the great truth that in a national system of education, if it is to be worthy of that name, it is the right of every pupil to receive instruction suited to his requirements, whether he be dull or not. But with the introduction of secondary education as part of the national system there is danger, Mr. Smith asserts, of a reintroduction of what is a virtue in private tuition, but a vice in a national system, namely individual teaching. There is, he says, a strong tendency on the part of many teachers to spend their time too exclusively upon their more brilliant pupils, and he mentions having visited one school where there was an accomplished and highly paid secondary staff who were practically engaged in the private tuition of a few of the smarter lads, the rest being dealt with in such a manner that it would have benefited them far more if they had been out in the playground or doing drill under the instruction of the drill-master.

We confess to a strong feeling of sympathy with Mr. Smith in what he says about the neglect of the "backwards"; nevertheless, we cannot but feel that the system which he deprecates is but a recognition of what, from a medical point of view, is of first importance in arranging for the education of children, namely, the extreme difference which exists both in physical and mental capacity between child and child. It is on this that is founded all that is now being done to provide special schools for those children who are defective in one direction or in another, and if we think it well to go to great expense in providing special instruction for those whose powers are deficient, and who will probably never give much return for all that they are taught, it does not seem unreasonable that special instruction should also be provided suited to those whose powers are in such excess that they would be practically wasting their time if taught along with average children. Such a proceeding, in fact, seems only to be in accordance with Mr. Smith's own ideas of a national education, namely, that it should be "suited to the requirements" of each pupil. Whether it would not be still better to teach every child his three R's and have done with it—providing plentiful scholarships, but leaving all secondary education to the universities—is another matter. It is now too late, however, to discuss that.

